# Supplementary material for: Mapping the evolution of fertility support policies in China: A content and instrumental analysis
Source: PLoS One. 2025 Oct 9;20(10):e0332137. doi: 10.1371/journal.pone.0332137 (PMC12510515; doi:10.1371/journal.pone.0332137)
Supplement: S1 Appendix — (ZIP) [file pone.0332137.s001.zip › S1 Appendix. 226 original policy documents/119-商务部等12部门关于推进城市一刻钟便民生活圈建设的意见(FBM-CLI-4-5013355).doc]

商务部等12部门关于推进城市一刻钟便民生活圈建设的意见

**发布部门：** [商务部](https://www.pkulaw.com/law/chl?Aggs.IssueDepartment=60220) [国家发展和改革委员会(含原国家发展计划委员会、原国家计划委员会)](https://www.pkulaw.com/law/chl?Aggs.IssueDepartment=60301) [民政部](https://www.pkulaw.com/law/chl?Aggs.IssueDepartment=60208) [财政部](https://www.pkulaw.com/law/chl?Aggs.IssueDepartment=60210) [人力资源和社会保障部](https://www.pkulaw.com/law/chl?Aggs.IssueDepartment=60236) [自然资源部](https://www.pkulaw.com/law/chl?Aggs.IssueDepartment=60243) [住房和城乡建设部](https://www.pkulaw.com/law/chl?Aggs.IssueDepartment=60238) [文化和旅游部](https://www.pkulaw.com/law/chl?Aggs.IssueDepartment=60241) [国家税务总局](https://www.pkulaw.com/law/chl?Aggs.IssueDepartment=60401) [国家市场监督管理总局](https://www.pkulaw.com/law/chl?Aggs.IssueDepartment=60426) [中国银行保险监督管理委员会](https://www.pkulaw.com/law/chl?Aggs.IssueDepartment=60322) [国家邮政局](https://www.pkulaw.com/law/chl?Aggs.IssueDepartment=7060342) [机构沿革](javascript:void(0))

**发文字号：**商流通函〔2021〕176号

**发布日期：**2021.05.28

**实施日期：**2021.05.28

**时效性：** [现行有效](https://www.pkulaw.com/law/chl?Aggs.TimelinessDic=01)

**效力级别：** [部门规范性文件](https://www.pkulaw.com/law/chl?Aggs.EffectivenessDic=XE0302)

**法规类别：** [商贸物资综合规定](https://www.pkulaw.com/law/chl?Aggs.Category=07501) [营商环境优化](https://dobusiness.pkulaw.com/)

商务部等12部门关于推进城市一刻钟便民生活圈建设的意见
（商流通函〔2021〕176号）

各省、自治区、直辖市、计划单列市及新疆生产建设兵团商务、发展改革、民政、财政、人力资源社会保障、自然资源、住房和城乡建设、文化和旅游、市场监管、邮政管理部门，国家税务总局各省、自治区、直辖市、计划单列市税务局，各银保监局：
　　一刻钟便民生活圈（以下简称便民生活圈），是以社区居民为服务对象，服务半径为步行15分钟左右的范围内，以满足居民日常生活基本消费和品质消费等为目标，以多业态集聚形成的社区商圈。近年来，各地便民生活圈快速发展，但也存在商业网点布局不均、设施老旧、新业态新技术新模式发展不平衡不充分等问题，亟待加以解决。为深入贯彻党的十九届五中全会和中央经济工作会议精神，坚定实施扩大内需战略，畅通国民经济循环，满足人民日益增长的美好生活需要，现就推进城市一刻钟便民生活圈建设工作提出如下意见。
　　**一、**总体思路
　　以习近平新时代中国特色社会主义思想为指导，把握新发展阶段，贯彻新发展理念，构建新发展格局，坚持以人民为中心的发展思想，按照试点先行、以点带面、逐步推开的思路，建立部、省、市、区、街道联动机制，以城市为实施主体，充分调动地方积极性，推动科学优化布局、补齐设施短板、丰富商业业态、壮大市场主体、创新服务能力、引导规范经营，提高服务便利化、标准化、智慧化、品质化水平，将便民生活圈打造成为促进形成强大国内市场、服务保障民生、推动便利消费及扩大就业的重要平台和载体。

　　**二、**基本原则
　　坚持以人民为中心。把实现人民对美好生活的向往作为奋斗目标，把人民满意作为工作的出发点和落脚点，发挥便民生活圈便民利民惠民的重要作用，不搞面子工程和形象工程。
　　坚持因地制宜。发挥政府引导、市场主导作用，遵循市场规律，不搞“一刀切”，聚焦补短板、堵漏洞、强弱项，推动设施配套化、服务多元化，不断提高服务水平，满足不同群体的消费需求，提升居民消费体验。
　　坚持系统观念。处理好整体与局部、发展与环境的关系，推动便民生活圈商业网点科学布局，商业设施与公共设施联动，商业运营与社区治理贯通，业态发展与居民需求匹配。
　　坚持守正创新。回归商业本质，以满足消费需求为核心，在提升实体商业服务质量与水平的同时，充分利用新技术推动业态和模式创新，线上线下融合，满足即期消费，激发潜在消费。

　　**三、**主要任务
　　（一）科学优化布局。加强统筹设计，顺应消费升级趋势，围绕便利消费、便民服务，明确便民生活圈商业网点规模、布局、业态结构和服务功能，与居民数量、消费习惯、经济水平等要素相适应。合理优化网点，重点对城镇老旧小区、新建居住区、城乡结合部小区加强商业网点布局，满足居民便利生活和日常消费需求。推动商居和谐，落实相关规划和标准，引导住宅和商业适当分离，商业设施和社区风格相协调，基本保障业态和品质提升业态相结合。
　　（二）补齐设施短板。结合城乡社区服务体系建设、城镇老旧小区改造等，开展广泛调研和排查，摸清底数，制定方案，明确新建和改造提升项目，推动便民商业设施进社区，打通“最后一公里”。支持盘活分散的社区空间资源，因地制宜配齐商业设施，通过标准化改造提升质量标准、环境卫生、服务品质。支持智能信包箱（快件箱）、箱式移动早餐售卖车、蔬菜直通车等便利设施进社区。支持有条件的社区改造提升商业中心、邻里中心等各类综合服务设施，完善“一站式”便民服务功能。鼓励有条件的物业服务企业向养老、托育、家政、邮政快递、前置仓等领域延伸，推动“物业服务＋生活服务”，提升消费便利化、品质化水平。
　　（三）丰富商业业态。鼓励商业与物业、消费与生活、居家与社区等场景融合，实现业态多元化、集聚化、智慧化发展。优先配齐基本保障类业态，支持与居民日常生活密切相关的便利店、综合超市、菜市场、生鲜超市（菜店）、早餐店、美容美发店、洗染店、药店、照相文印店、家政服务点、维修点、再生资源回收点、邮政快递综合服务点、前置仓等进社区，在安全、合法的前提下采取“一点多用”、服务叠加等方式发展微利业态，保障生活必需。因地制宜发展品质提升类业态，鼓励发展特色餐饮、运动健身、保健养生、新式书店、教育培训、休闲娱乐、老年康护、幼儿托管等品质提升类业态，促进商文旅融合，拓展社交化、特色化功能，满足居民多样化的消费需求，提升生活品质。
　　（四）壮大市场主体。发挥市场在资源配置中的决定性作用，激发各类市场主体活力，促进集聚式发展。鼓励大企业输出品牌、标准、管理和服务，发展社区便利店、超市、理发店、餐饮店（早餐、快餐）等直营连锁，对形象标识、门店管控、设施配置、服务标准、商品采购、物流配送实行“六统一”。鼓励企业以大带小，在做好自营商超的同时，开放供应链、物流渠道及门店资源，为传统夫妻店、杂货铺提供集采集配、统仓统配等一站式服务，通过商业特许经营方式实现品牌化、规范化发展。支持菜市场、生鲜超市（菜店）标准化改造，推广先进冷链技术和设施设备，加强供应链管理，实行超市化、连锁化经营。鼓励数字化赋能，引导第三方技术服务商把大数据应用到开店布局、进销存管理、物流配送、商品追溯等各个环节，促进模式创新和市场要素精准配置。推动平台企业为高质量发展和高品质生活服务，坚持发展与规范并重，为社区商户提供营销、信息、流量、数字化工具等免费或让利服务，将实体店作为供应链合作的一环，实现线上线下融合、店配宅配融合，加快配置社区团购必备的冷链等设施设备，健全管理制度，保障食品安全和公众利益。
　　（五）创新服务能力。增强服务便利，鼓励“一店多能”，搭载代扣代缴、代收代发、上门服务、租赁等项目，通过跨界经营提高便民服务能力。提升服务品质，推广专业化托管、连锁经营、农超对接、店仓配一体等成熟模式，引导差异化、特色化经营，提高管理效能和服务供给能力。拓展智能体验，鼓励应用5G、大数据、人工智能、物联网等先进信息技术，驱动发展无接触交易、智能结算、网订店取（送）、直播带货、自助售卖等创新模式，拓展便民生活圈应用场景。优化信息服务，支持依托智慧社区信息系统，构建城市便民生活圈智慧服务平台（小程序或APP），整合本地商户资源，接入购物、餐饮、休闲、文化、养老、托育、家政等线上功能，提供周边商品和服务搜索、信息查询、生活缴费、地理导航及线上发券、线下兑换等免费服务，打造集约式发展生态圈，推动接入智慧城市和基层管理服务平台。
　　（六）引导规范经营。发挥商协会作用，鼓励制定相关标准，开展技能培训，规范商户经营和服务行为。完善管理制度，整合街道、社区、物业、商户等各方力量，强化诚信经营和守法意识，通过共建共管加强环境整治和自律规范。引入专业运营商，鼓励整体规划、统一招商、统一运营、规范管理，完善购物、服务、休闲、健身、文化、社交等功能。照顾特殊群体，完善必要的无障碍设施，张贴便民生活圈导视图，鼓励设立为老年人服务的专柜和体验店，保留实体店铺面对面人工服务，支持现金和银行卡支付，提供简便易行、满足老年人基本需要的服务方式，提高便民服务的“温度”。

　　**四、**保障措施
　　（一）加强组织领导。
　　各地结合实际把便民生活圈建设纳入政府保民生重点工程，作为实施扩大内需战略和做好“六稳”工作、落实“六保”任务的有效手段。鼓励实行市长（区长）负责制，加强顶层设计，制定城市便民生活圈建设专项规划，在用地、用房、财政、金融、营商环境等方面加大政策扶持力度，重点解决居民消费难点痛点问题。推动开展城市一刻钟便民生活圈建设试点，加强部门联动，商务、住房和城乡建设主管部门重点加强工作指导，发展改革、民政、财政、人力资源社会保障、自然资源、文化和旅游、税务、市场监管、金融、邮政管理等部门要发挥职能作用，落实支持政策，加强监督管理，优化营商环境，共同推动便民生活圈健康发展。（有关部门，各地方人民政府负责）
　　（二）强化政策保障。
　　落实新建社区商业和综合服务设施面积占社区总建筑面积比例不低于10%的规定，做到社区商业设施与住宅同步规划、同步建设、同步验收和同步交付。结合旧城改造和城市更新，做好与国土空间规划衔接，推动土地复合开发利用、用途合理转换，盘活存量房屋设施，增加商业网点用房供给，有条件的商业网点周边要实行人车分流，完善无障碍设施。鼓励物业服务企业在保障安全、征得业主同意的前提下，在居住区设置共享仓，为商户和居民提供物品临时存放场所。（民政部、自然资源部、住房和城乡建设部按职责分工负责）
　　鼓励共享办公、线上线下融合等新模式新业态新服务发展，加大支持微利业态经营的力度，清理规范用电环节不合理加价行为；鼓励各地结合实际制定房租减免、补贴政策，推动降低社区店铺经营成本。（发展改革委、住房和城乡建设部、商务部、市场监管总局按职责分工负责）
　　落实创业担保贷款贴息政策。支持地方用好就业补助资金，对符合条件的商户按规定落实就业补贴政策，对符合条件的企业按规定给予失业保险稳岗返还。（财政部、人力资源社会保障部按职责分工负责）
　　引导银行保险机构加大普惠金融支持力度，对行业主管部门推荐的为老服务、应急保供等名单企业（含信用信息）依法依规提供信贷、保险优惠政策，创新消费信贷服务，为社区提供专业、便捷、贴心的金融服务。（银保监会负责）
　　把智能信包箱（快件箱）纳入社区公共服务设施建设。（住房和城乡建设部、国家邮政局，各地方人民政府负责）
　　鼓励有条件的地方对便民生活圈商户购买的财产险、食品安全险、职业责任险等适当补贴，提高商户经营的抗风险能力。（各地方人民政府负责）
　　落实国家关于养老、托育、家政等社区家庭服务业税费优惠政策。落实生活性服务业增值税加计抵减及普惠性减税降费政策。落实推动品牌连锁便利店加快发展的有关政策。（民政部、财政部、商务部、税务总局按职责分工负责）
　　（三）优化营商环境。
　　持续深化商事制度改革，优化企业开办服务，推广电子证照应用，支持各地加大住所与经营场所登记改革力度。实施“包容审慎”监管，坚持处罚与教育相结合，对违法情节轻微、无违法后果或后果较轻的，在法律法规允许的幅度范围内给予适当从轻、减轻或免予处罚，指导和帮助市场主体整改。（市场监管总局，各地方人民政府负责）
　　简化社区店铺开业程序，装修施工、招牌设置实行备案承诺制，公众聚集场所投入使用、营业前消防安全检查实行告知承诺制。（各地方人民政府负责）
　　坚持发展和规范并重，建立健全社区电商（含社区团购等）领域市场准入规则，划清底线、加强监管、规范秩序，促进公平竞争，反对垄断，防止资本无序扩张。加强规范和监管，维护公众利益和社会稳定，督促平台企业承担商品质量、食品安全保障等责任，落实社区团购“九不得”规定，维护线上线下公平竞争的市场环境。畅通投诉举报渠道，完善消费纠纷解决机制和消费者反馈评价机制。（商务部、市场监管总局按职责分工负责）
　　（四）夯实工作基础。
　　鼓励第三方机构为社区小微企业和个体工商户提供数字化转型、信息咨询、装修设计、营销策划、经营分析等专业服务。鼓励商协会建立新商业模式成熟度评估制度，加强标准制修订，联合职业学校等多渠道开展线上线下培训，深化校企合作，提升从业人员能力。完善统计制度，加强便民生活圈的店铺数量、从业人数、居民满意度、建设改造投资额等指标统计。创新工作宣传和交流方式，把居民满意不满意作为检验标准，边试点、边总结、边推广成熟经验做法，不断增强人民群众获得感、幸福感、安全感。（各级商务部门委托有关协会、社会机构负责）
　　联系方式：商务部流通发展司
　　任宏伟010-85093794，罗旻慧010-85093777
　　010－85093767（传真），luominhui@mofcom.gov.cn
　　附件：城市一刻钟便民生活圈建设试点方案

商务部
　　发展改革委
　　民政部
　　财政部
　　人力资源社会保障部
　　自然资源部
　　住房和城乡建设部
　　文化和旅游部
　　税务总局
　　市场监管总局
　　银保监会
　　邮政局
　　2021年5月28日

　　附件

　　城市一刻钟便民生活圈建设试点方案

　　为贯彻落实《关于推进城市一刻钟便民生活圈建设的意见》，推动实施扩大内需战略，加快城市一刻钟便民生活圈建设（以下简称便民生活圈），畅通国民经济循环，满足人民日益增长的美好生活需要，今年起拟开展城市便民生活圈建设试点，现将有关事项通知如下：

　　**一、**试点工作目标
　　“十四五”时期，在全国选择条件成熟的城市开展便民生活圈试点，指导建设一批布局合理、业态齐全、功能完善、智慧便捷、规范有序、服务优质、商居和谐的便民生活圈，通过全国推广经验，构建便民生活圈、打造便民服务链、提升民生幸福度，切实增强人民群众获得感、幸福感、安全感。

　　**二、**试点城市申报原则和条件
　　（一）政府重视，积极性高，发展基础好，实施方案科学。
　　（二）推动深化“放管服”改革、优化营商环境，已落实财税、信贷、就业等支持政策。
　　（三）前期已开展便民生活圈或社区商业相关工作，出台有配套支持政策，取得成熟经验和阶段性成效的城市；实行“市长（区长）负责制”的城市；已纳入省级试点的城市等，将优先纳入全国性试点。

　　**三、**申报流程和时限
　　申报流程：城市是试点申报主体，实施方案等申报资料由城市人民政府盖章后，上报至省级商务主管部门；省级商务主管部门是推荐主体，商有关部门同意后，由省级商务主管部门盖章上报至商务部，商务部（流通发展司）、住房和城乡建设部（建筑节能与科技司）会同有关部门负责试点具体工作。
　　“十四五”时期，省、自治区每年推荐不超过2个城市（省会、地级），直辖市、新疆生产建设兵团每年推荐不超过2个区或师；计划单列市政府可直接申报。请各省级商务主管部门于2021年7月15日前推荐首批试点申报城市。

　　**四、**试点内容要求
　　按照《关于推进城市一刻钟便民生活圈建设的意见》要求，以城市为试点实施主体，重点围绕科学优化布局、补齐设施短板、丰富商业业态、壮大市场主体、创新服务能力、引导规范经营等开展试点，探索并推广在政策创新、制度创新、管理创新、模式创新等方面的经验，逐步扩大便民生活圈覆盖范围。
　　试点城市应结合本地实际，科学制定实施方案，做到思路清晰、目标量化、任务具体、数据真实、措施有效、特色突出、图文结合。城市申报方案思路应体现“两年试点、三年推广”，方案内容应包括：发展现状和问题，工作思路，逐年工作目标（每年地级城市及直辖市的区至少5个生活圈、省会及计划单列市至少10个生活圈，并列出覆盖不同类型便民生活圈的名单），主要任务，保障措施，责任分工，试点项目进度安排表。方案、附表、地方政策文件等材料，请一并上报。

　　**五、**工作职责分工
　　（一）国家层面
　　商务部、住房和城乡建设部会同有关部门负责工作统筹、宏观指导、政策研究，每年选取不同类型的城市开展试点，在政策创新、制度创新、管理创新、模式创新等方面进行探索，总结推广成熟可复制的经验做法；委托各省级商务、住房和城乡建设主管部门会同有关部门分批对试点城市整体情况进行评估，并抽查评估结果，以适当方式在一定范围内公布。
　　（二）地方层面
　　省级商务、住房和城乡建设主管部门会同有关部门负责业务指导和跟踪督促，协调支持政策，推荐全国性试点城市，探索开展省级试点，对承担全国性和省级试点的城市完成任务整体情况进行分批评估，并在全省范围内及时总结推广经验。
　　城市是试点实施主体，实行市长（区长）负责制；城市商务、住房和城乡建设主管部门会同有关部门组织试点，因城施策、因地制宜，灵活采用网络问卷、座谈会、现场调研等方式，问需于民、问计于民、问政于民，摸清现状和问题，理清工作思路，编制城市便民生活圈建设专项规划，制定实施方案、支持政策，按照问题导向、目标导向加强项目管理和验收；建立工作考核和奖励机制，调动区、街道各级工作积极性，做到与民生保障相结合、与消费升级相结合、与发展新模式新业态相结合、与畅通国民经济微循环相结合，通过“两年试点、三年推广”，确保取得扎实成效。
　　附：1.城市便民生活圈建设试点情况汇总表
　　2.试点便民生活圈建设情况调查表
　　附1
　　城市便民生活圈建设试点情况汇总表
　　试点城市： 截至日期：

| 城市社区总数量（个） | |  | 上年底城市常住人口（万人） | | | | |  | | 上年底城市社会消费零售总额（亿元） |  | |
| --- | --- | --- | --- | --- | --- | --- | --- | --- | --- | --- | --- | --- |
| 试点社区生活圈的类型划分 | | | | | 试点社区有关情况 | | | | | | | |
| 集聚式为主（个） | 街坊式为主（个） | 分布式为主（个） | 小计 | 品牌连锁店数量（个） | | | 品牌连锁数量占社区商业网点数量比例（%） | 商业中心和邻里中心数量（座） | 商业网点从业人数（万人） | 服务社区的居民数量（万人） | | 备注 |
|  |  |  |  |  | | |  |  |  |  | |  |
| 试点社区的商业网点（店铺）按业态分类统计 | | | | | | | | | | | | |
| 合计 | 基本生活类商业网点数量（个） | | | | | | | | | 提质生活类商业网点数量（个） | | |
|  | 品牌连锁便利店 | 综合超市 | 菜市场 | 生鲜店（菜店） | | 早餐店 | 维修点 | 其他店铺 | 养老站 | 新式书店 | 运动健身房 | 其他店铺 |
|  |  |  |  |  | |  |  |  |  |  |  |  |
| 年度 | 试点推进 | 逐年推进试点的社区数量（个） | 建设改造社区商业中心（邻里中心）数量（座） | 新增和改造商业网点（店铺）数量（个） | | | 其中新增和改造品牌连锁店数量（个） | 新增带动就业人数（人） | 各级政府财政投资额（万元） | 社会投资额（万元） | 便民生活圈智慧服务平台应用的社区数量（个） | 试点社区居民对商业网点的综合满意度（％） |
| 2021年 | 计划 |  |  |  | | |  |  |  |  |  |  |
|  | 已完成 |  |  |  | | |  |  |  |  |  |  |
| 2022年 | 计划 |  |  |  | | |  |  |  |  |  |  |
|  | 已完成 |  |  |  | | |  |  |  |  |  |  |
| 2023年 | 计划 |  |  |  | | |  |  |  |  |  |  |
|  | 已完成 |  |  |  | | |  |  |  |  |  |  |
| 2024年 | 计划 |  |  |  | | |  |  |  |  |  |  |
|  | 已完成 |  |  |  | | |  |  |  |  |  |  |
| 2025年 | 计划 |  |  |  | | |  |  |  |  |  |  |
|  | 已完成 |  |  |  | | |  |  |  |  |  |  |
| 说 明 | 1.此表为汇总表，由城市填写，为方便汇总请勿改表格结构。计量单位均保留小数点后两位。 2.居民满意度指标：每个社区抽样调查不少于50份。 | | | | | | | | | | | |

　　附2
　　试点便民生活圈建设情况调查表
　　试点社区名称： 所在城市： 截至日期：

| 运营主体（多选，打√） | | 街道 国企 民营企业 专业运营商 网点各自经营 另：是否有便民生活圈智慧服务平台 | | | | | 发展形态 | 集聚式为主 街坊式为主 分布式为主 | | |
| --- | --- | --- | --- | --- | --- | --- | --- | --- | --- | --- |
| 社区商业中心（邻里中心）数量（座） | | | |  | 其中新增和改造数量（座） | |  | 服务社区的居民数量（万人） | |  |
| 分类 | 主要业态网点 | 商业网点（店铺）数量合计（个） | 从业人数（万人） | 其中 | | | 各级政府财政投资补助额（万元） | 社会投资额（万元） | 居民满意度（%） | 备注 |
|  |  |  |  | 品牌连锁店数量（个） | 新建和改造网点（店铺）数量（个） | 新增从业人员数量（个） |  |  |  |  |
| 合 计 | |  |  |  |  |  |  |  |  |  |
| 基本保障类 | 便利店 |  |  |  |  |  |  |  |  |  |
|  | 综合超市 |  |  |  |  |  |  |  |  |  |
|  | 菜市场 |  |  |  |  |  |  |  |  |  |
|  | 生鲜超市（菜店） |  |  |  |  |  |  |  |  |  |
|  | 早餐店 |  |  |  |  |  |  |  |  |  |
|  | 维修点 |  |  |  |  |  |  |  |  |  |
|  | 洗染店 |  |  |  |  |  |  |  |  |  |
|  | 美容美发店 |  |  |  |  |  |  |  |  |  |
|  | 照相文印店 |  |  |  |  |  |  |  |  |  |
|  | 药店 |  |  |  |  |  |  |  |  |  |
|  | 家政服务 |  |  |  |  |  |  |  |  |  |
|  | 邮政快递综合服务点（快件箱组） |  |  |  |  |  |  |  |  |  |
|  | 再生资源回收点 |  |  |  |  |  |  |  |  |  |
|  | 其他 |  |  |  |  |  |  |  |  |  |
| 品质提升类 | 新式书店 |  |  |  |  |  |  |  |  |  |
|  | 运动健身 |  |  |  |  |  |  |  |  |  |
|  | 特色餐饮店 |  |  |  |  |  |  |  |  |  |
|  | 养老站 |  |  |  |  |  |  |  |  |  |
|  | 托育站 |  |  |  |  |  |  |  |  |  |
|  | 保健养生 |  |  |  |  |  |  |  |  |  |
|  | 教育培训 |  |  |  |  |  |  |  |  |  |
|  | 休闲娱乐 |  |  |  |  |  |  |  |  |  |
|  | 其他 |  |  |  |  |  |  |  |  |  |
| 说明 | 1.此表为调查明细表（与汇总表相对应），由街道（社区）填写，为方便汇总请勿改表格结构。计量单位均保留小数点后两位。 2.居民满意度指标：每个社区抽样调查不少于50份。 | | | | | | | | | |

© 北大法宝：（[www.pkulaw.com](https://www.pkulaw.com/)) 专业提供法律信息、法学知识和法律软件领域各类解决方案。北大法宝为您提供丰富的参考资料，正式引用法规条文时请与标准文本核对。
欢迎查看所有[产品和服务](https://www.pkulaw.com/helps/2.html)。法宝快讯：[如何快速找到您需要的检索结果？](https://www.pkulaw.com/helps/70.html)[法宝](https://www.pkulaw.com/helps/69.html)V6有何新特色？

扫描二维码阅读原文

**原文链接：**<https://www.pkulaw.com/chl/c366824182b5b379bdfb.html>
